# Challenges and Opportunities: What Can We Learn from Patients Living with Chronic Musculoskeletal Conditions, Health Professionals and Carers about the Concept of Health Literacy Using Qualitative Methods of Inquiry?

**DOI:** 10.1371/journal.pone.0112041

**Published:** 2014-11-06

**Authors:** Charlotte Salter, Julii Brainard, Lisa McDaid, Yoon Loke

**Affiliations:** Norwich Medical School, Faculty of Medicine & Health Sciences, University of East Anglia, Norwich, NR4 7TJ, United Kingdom; The University of York, United Kingdom

## Abstract

The field of health literacy continues to evolve and concern public health researchers and yet remains a largely overlooked concept elsewhere in the healthcare system. We conducted focus group discussions in England UK, about the concept of health literacy with older patients with chronic musculoskeletal conditions (mean age  = 73.4 years), carers and health professionals. Our research posed methodological, intellectual and practical challenges. Gaps in conceptualisation and expectations were revealed, reiterating deficiencies in predominant models for understanding health literacy and methodological shortcomings of using focus groups in qualitative research for this topic. Building on this unique insight into what the concept of health literacy meant to participants, we present analysis of our findings on factors perceived to foster and inhibit health literacy and on the issue of *responsibility* in health literacy. Patients saw health literacy as a result of an inconsistent interactive process and the implications as wide ranging; healthcare professionals had more heterogeneous views. All focus group discussants agreed that health literacy most benefited from good inter-personal communication and partnership. By proposing a *needs-based* approach to health literacy we offer an alternative way of conceptualising health literacy to help improve the health of older people with chronic conditions.

## Background

Health literacy (HL) is an evolving concept [1]. Definitions and practical intervention models vary widely [2]–[5]. The most common perspective is that HL is a personal asset, a package of competencies relevant to personal healthcare, and a skill-set typically ascribed mostly or solely to individual patients rather than to context or healthcare delivery systems. Estimates are that 15–26% of adults in developed nations have poor HL, while a further 20–29% have HL skills that are at best ‘problematic’ or ‘marginal’ [6]–[8]. HL is measured as poorest in the oldest age cohorts. This may be due to fewer years of formal education amongst this age group, increased complexity of healthcare and medical needs and/or decline in cognitive function [9].

This paper discusses methodological challenges in using focus groups to undertake qualitative research into HL in older adults. We explore how the HL concept is interpreted by patients, their carers and health professionals, and how these interpretations may differ from predominant research directions and academic models. We propose that HL might be more accurately described as a ‘whole-system’ construction rather than as either an individual asset (something that can be promoted) or an individual risk (something that is missing and needs correcting). This perspective seems to ally more with the minority model of HL as a risk, arising from a set of attributes belonging to patient, setting, modes of delivery and other provider features [3], [4]. However, much focus in the field of HL at the risk level concentrates on the notion of individual time and place specific clinical risk and only measured cross-sectionally. The emergence of a broader conceptualisation of a risk model of HL in patients' understanding allows us to revisit shortcomings in both the traditional asset and risk models. We explore some of the limitations in previous interpretations of the risk view of HL, and how it might be redeveloped and perhaps even integrated with the asset model. The contrast between an asset model and a whole-system attributes approach is significant and has many implications for healthcare delivery. However, the observations are framed by the characteristics of the study participants and to some extent by the data collection techniques. The research was also undertaken within the context of how health care is usually obtained in the UK, which is through the National Health Service (NHS), a taxpayer funded service which provides the vast majority of medical care in the UK. We therefore present our suggestions in the context of methodological challenges in undertaking such qualitative research.

## Introduction

Early (1990s) definitions and conceptual models of health literacy (HL) were concerned with identifying patients with significant shortcomings in literacy and numeracy skills. These basic deficits were observed to comprise >40% of US adults unable to read complex text [10] and one in five UK adults with less literacy than expected of an 11 year old child and up to 40% of adults with significant numeracy problems [11]. It was anxiety raised by these statistics that fuelled an interest in health literacy where in the context of the US and UK healthcare systems both rely extensively on complex written and verbal information to guide patients. Nutbeam (2000) [12] categorised HL into 3-levels: functional, interactive and critical. Functional HL is concerned with basic skills (reading and numeracy). Interactive HL further involves communicative and social skills necessary to understand and apply new information in changing situations. Critical HL is a ‘higher level’ set of cognitive and social skills used to critically examine health information. Chinn [13] described critical HL as a social asset (whereas functional HL may be seen as a health knowledge deficit). Numerous assessment instruments have been developed to measure functional and sometimes interactive HL in individuals [14], [15]. Most such instruments are relatively easy to administer but are quite simplistic in what they assess, focusing primarily on word or short sentence comprehension. Much research exists that establishes a consistent deleterious association between low HL levels as indicated by those instruments and health outcomes [16], [17] although the full causal pathways remain uncertain [16]–[18].

The view of HL as an individual attribute (or asset) has dominated and indeed is the basis of many intervention and health promotion efforts. However, variations on the perspective have emerged. Baker [19] described HL as an individual's ability to function in a healthcare environment, while Volandes and Paasche-Orlow [20] argued that HL can only be addressed and understood in the context of other health-related inequalities. Nutbeam (2008) [4] described Baker's work as a ‘risk’ model of HL and critiqued it as too limited in scope for development of interventions but welcomed perspectives which might better integrate the risk and assets models. Most practical guidance to address poor HL (e.g. Nielsen-Bohlman et al. [10]) has stated that while HL levels are individual patient assets, health professionals have joint responsibility with patients for tackling problems of low HL. Other recent definitions and models [2], [21] describe HL as an individual or population asset, while simultaneously portraying it as a product of both context (including external mediators) and patient capacities. Common to all such research is an untested underlying assumption that if people are given the conditions (skills, awareness, information etc) to make individual informed decisions, then they will ‘do the right thing’: i.e. adopt public health and (bio)medical strategies deemed necessary and important to promote and maintain good health. As a result, poor health literacy has become almost synonymous with undesirable health choices. We will return later to the tangled problems of low HL and non-adherence.

## Methods

This research was part of a National Institute for Health Research study to evaluate the impact of low HL on older people with chronic conditions and identify areas for improvement in patient care and future research. Musculoskeletal conditions were chosen because we had conducted a systematic review and collected published data on these conditions. The specific aim of the focus group discussions (FGD) was to explore with older people, carers and healthcare professionals the concept of HL and issues they believed might impact on the HL of older people with musculoskeletal conditions. All FGD took place in 2012 in the East of England. Ethical approval was granted by East of England National Research Ethics Committee – 09/H0310/30.

We conducted six interactive focus groups with older patients living with musculoskeletal conditions, carers and their health professionals (HP) recruited from secondary care, primary care and community settings run by the National Health Service (NHS). See Table 1. We employed a three-prong approach to recruitment as we envisaged it might be challenging. In the event three focus groups were conducted (n = 15) with older patients (mean age  = 73.4 years) recruited through the local hospital rheumatology department; a community support group for people with rheumatoid conditions, and via a specialist rheumatology nurse at a large primary care medical centre. Prominent posters advertised the research and staff were asked to give information packs to suitable patients. Although we aimed for a purposive sample to represent a broad a range of characteristics including social class, gender and education, recruitment was challenging and we became reliant on HP to give information packs to potentially eligible consecutive patients with at least one long-term musculoskeletal condition. The researcher (LM) then followed up potential participants with a phone call to explain the study in full and ask if they were willing to take part. Everyone approached agreed to take part although at least one person dropped out on the day of each of the patient focus groups (n = 5). Rheumatoid arthritis was the main chronic illness reported by patients (n = 10), with four other musculoskeletal conditions and type II diabetes also represented. All bar one patient considered their health to be fair to good at the time of the FGDs. Two thirds of participants were female. We achieved a range of socio-economic backgrounds. Nearly two thirds left school by age 15 and three went on to higher education. All but one gave their current employment status as retired. Focus group discussions were held at the university research park and the primary care medical centre respectively.

**Table 1:** Focus Group Participants by Recruitment Site.

Group Number	Type of participant	No of participants
FG1	Patients recruited in primary care setting	5
FG2	Patients recruited in community Group	6
FG3	Patients recruited in hospital setting	5
FG4	Carers recruited in primary care setting	2
FG5	Health professionals in primary care	11
FG6	Health professionals in hospital setting	5

Two focus groups were conducted with HPs (n = 16); one in secondary care and one in primary care. See Table 1. Details of individual participants are not included in order to maintain confidentiality. The primary care focus group included 6 general practitioners, two of whom were male, plus five nurse practitioners. The hospital based focus group was an all-female group that consisted of five members of the specialist practitioner multidisciplinary rheumatology team including nurses, a healthcare assistant and occupational therapists but no physicians. Unfortunately, recruitment of carers proved difficult, despite over a thousand leaflets being distributed within a local charity newsletter (Norwich Age UK). The leaflet asked ‘can you help improve services for older people living with chronic illnesses' and carers were specifically invited to attend a group discussion about accessing, using and understand health care services. Travel and replacement carer costs plus refreshments were offered to all. We eventually recruited 2 carers (only) via an existing carers support group at the same primary care medical centre and had another carer in attendance at a patient FGD to support her husband's personal care needs and speak for him when he got exhausted during the discussion. A fourth potential carer sadly declined to participate after he found the consent forms too stressful to fill in. Recently bereaved, he had ‘had enough of red tape and paper work to last a lifetime’. This highlighted to us the excess burden that constant form filling and applications for support services can place on vulnerable people.

### Data collection

A topic guide was used to ensure the same domains were covered in each focus group and open-ended questions and prompts used to encourage participants to talk about their own experiences and views as well as those of their peers. Domains included: i) understanding of the concept of HL and how it might impact on the experience of older people living with long term chronic health conditions; ii) experiences of accessing, navigating and engaging with the healthcare system; and, iii) factors that might enhance or impede HL. We aimed not to impose any existing theoretical framework on the discussions. The focus group discussions lasted average 81 minutes (range 72–91) and were interactive as after approximately one hour ‘trigger material’ was presented to participants to stimulate further discussion. This took the form of a short 2 minute video clip about health literacy with Professor Rima Rudd talking about how words can get in the way of understanding and access to health (https://www.youtube.com/watch?v=_d-dtYTpdCw) and a brief summary of our findings from the literature about the issues facing older people with low HL and chronic conditions [22], [23]. FGD were audio recorded, transcribed verbatim and all identifiers removed.

Two experienced moderators (CS and LM) were present at each FGD and reassurance given that the views of all participants would be respected equally and all identifiers removed from transcripts and subsequent documentation. All groups aimed to maintain a friendly and approachable (casual and non-intimidating) atmosphere and moderators invited contributions from all participants. The HP were known to one another and talked informally and openly. Some of the patients and carers knew one another by sight through support groups. Travel expenses and replacement carer expenses were offered and refreshments were provided. All participants gave written consent and completed a simple baseline questionnaire including information on age, gender, educational attainment, subjective well-being and chronic conditions experienced. Although females predominated in all 5 focus groups the male patients were equal contributors. However, the two male GPs were more reticent than their female clinical and nursing colleagues. Respondents were offered a copy of their transcript for the purpose of checking they were still happy for us to use their contribution. Findings were fed back to a multidisciplinary group of health professionals (HP) at an education meeting at the regional hospital.

### Data analysis

A thematic analysis was applied to the fully transcribed focus group transcripts. Familiarisation, data management, coding and categorisation were carried out by the three members of the interdisciplinary research team. Iteration within and between patient, carer and HP data sets and the research literature helped inform the analysis at the explanatory level. Participant attributes such as occupation and gender were mapped and considered during the analysis stage. The principles of framework analysis [24] were used to order, chart and search the data both manually and supported by relevant software (NVivo 9 Software, MSWord and Framework). In particular, attempts were made in the beginning to map findings across existing models of HL but this was found to be difficult and ultimately inappropriate.

## Findings

Building from our starting point of exploring what the concept of HL meant to participants, we present the analysis of our findings under two broad themes: i) the meaning of health literacy for patients and carers, and health professionals; and, ii) health literacy, governance and responsibility. Study findings are presented as both extracts of participants' social interactions [25] and illustrative individual quotes. Extracts are labelled using participant pseudonym (first names for patients and carers, and surnames for HP) and focus group attended. All identifiers have been removed.

### i) The meaning of health literacy

#### Patients and carers

Discussion of the term ‘health literacy’ was problematic with only one participant across all six focus groups (Nurse Ford in FG6) having any prior knowledge of the concept. Patients and carers had some strong views about the term ‘health literacy’ with several feeling it was an unhelpful term, too much linked to formal learning and literacy. On several occasions in the non-HP groups participants tried to help each other out as in this exchange between two carers in FG4:

Diane: *I mean you earlier mentioned this word health literacy and I′ve actually tried to avoid using it because I think it's a very academic term which actually when you say to someone like yourself (Linda) or myself what does it actually mean? I suppose they′re now trying to refer to this area about how patients and carers use and understand health information and services and does it make sense to them*

Linda: *Well then understanding about your health like any other literacy uh ‘computer literate’ means you know how to use a computer so presumably (it's the same)*

When asked to state initial impressions of the term ‘health literacy’, typical responses from carers and patients concerned comprehension and understanding. These could be at both a very personal and intrinsic level where ill health hindered comprehension, and at a more extrinsic or practical level. This exchange between patients and a carer in FG3 illustrates both levels:

James: *I would like to understand more I must admit and uh that's the difficult bit I have various illnesses um I just can′t, sometimes it's completely gobbledygook*

Vivien: *I′m Vivien James's wife and um health literacy to me first of all means understanding how you can get appointments quickly, understanding the forms that come through, also understanding when doctors talk to you and consultants speak. Sometimes they speak very fast because they know what they′re speaking about, they use words that you′re not always used to and you′d like to very much be able to stop them in full flow and say ‘what is this’ ‘what do you mean?’ can you explain’?*

Jill: *I agree with Vivien largely but I think it means understanding my problems and being able to ask questions which over the years I′ve had my condition now for 18 years I do feel more confident to ask questions*

Being able to follow and adhere to doctor's instructions was another common way patients and carers interpreted the notion of health literacy for others. However for themselves, patients and carers cited the importance of knowing many ‘tricks’ of the system to more efficiently get their needs met. These tricks ranged from being able to get suitable appointments and ask questions through to the ability to interrogate information and do their own research. Joan and Barbara's in FG2 exchange examples of their expertise in note making:

Joan: *The other thing, after that consultation on Monday, as soon as I got out I wrote down everything she said so that when I go to see the GP in two weeks' time I will remember it*

Barbara: *Well I always write everything down as well because as you say you tend to get in there and you think oh what am I going to ask? So if I write it down I find that very helpful*

Many of our patients had become expert patients over years of living with their chronic conditions (mean  = 19 years; range 4–35) and routinely engaged with their health needs. These ‘tricks’ and skills that had developed over time illustrate increasingly sophisticated competencies (see Nutbeam 2008) and include the ability to interpret and critique healthcare information. Furthermore participants saw themselves as well informed about their own conditions and even able to support friends and family as Barbara again highlights here:

*I don′t know. Because if I I′ve got anyone close to me that's got something wrong with them I sort of do investigate. But I don′t feel your GP tell you exactly what you′ve got, and how you can cope with it. Is there any preventive cures etc?* (Barbara:FG2)

Patients and carers portrayed the attainment and maintenance of health literacy as an ongoing process. Many described their learning journeys including mistakes made by themselves or HP. Patients often voiced frustration that they were expected to rise to the expectations of the healthcare system and manage inadequate communication rather than the system adapting itself to meet their needs. They implied that for them personally, any lack of HL was a defect in the system, a system that did not give them, or help them, discover and develop the information or skills they need. Also, patients did not suggest that good HL was a one-sided attribute. They often described HL as the result of good two-way communication, particularly between patient and professional, with both sides needing to bring equal interest and skills to the relationship. However, patients were not surprised to be informed that measured HL is lowest among older adults. Many had anecdotes about older friends, family or neighbours who struggled with health management. They cited social isolation as a causal factor, and lack of awareness about choices or rights to access services. There was talk of how chronic illness undermines self-confidence and the risks of dementia were mentioned:

*I used to go to a bungalow where an old couple lived. She could hardly see at all. They were in their 80's. The chemist would deliver to them a box full of medication and there he was trying to sort his wife's out and his own out getting utterly mixed up and in fact he went by colour. So I used to go in and help him sort out what was what and how many times a day and so on [they were] totally incapable of sorting it.* (Jean:FG1)

#### Health Professionals

Health professionals were quick to grapple with the idea of HL and what it meant in their work. They also saw significant barriers in identifying HL levels including embarrassment and stigma, and described ‘gauging’ (but not directly asking about) patients understanding and need for information. Most HP had anecdotes about patients whose social needs or literacy shortcomings they had failed to recognise. HP were keen to provide healthcare information at the appropriate level of complexity, but noted it was difficult when patients might not be open if asked ‘can you read that okay’ as the following extract exemplifies:

*I had a little lady who nearly starved once. Her elderly brother used to look after her and it wasn′t [until] he died, that I realised that she couldn′t read. And she couldn′t shop, do you know what I mean? You forget you′ve made assumptions about people's literacy levels all the time.* (Dr Shelley:FG5)

Professionals (and patients) agreed that the best way to identify patient HL needs was by spending enough time talking to patients. HP could see deficits in their own approach to information giving as the following exchange in the primary care professional FG5 reveals. Here the doctors are aware that by comparison much of their own communication and institutional literature is poorly worded and targeted, especially when compared to the way in which the media was able to construct and target an idea:

Dr Gregory: *The literacy level of the information given doesn′t match the understanding level of the patient so you can blame the professional instead of the patient.*

Dr Jones: *[Newspapers] always pitch their literacy levels better. I mean that was always something you were aware of and I think they still do, which is why we get so many people coming forward with things that they have understood from their newspaper article but haven′t accessed other things that we probably provided.*

Dr Patel: *The moral of that is get them [journalists] to write the health information leaflets.*

Health professionals also identified difficulties in communicating information that went beyond their ability to present information in a clear and accessible manner. In particular they discussed the difficulties posed when patients struggled to understand or accept a diagnosis, or had personal views and preferences about their health management that would preclude acceptance of a diagnosis or treatment. Thus another integral part of health literacy communication was ensuring information is relevant as these exchanges in FG5 illustrate (including when a doctor becomes a patient himself):

Nurse Rowan: *The amount of people that are coming in post op and they don′t seem to know how much exercise they can do or when the dressing needs to be changed or … you think they must have been told and they must have had some written information.*

Nurse Castle: *Exactly*

Dr Jones: *But it's whether you think it's applicable to you as well. Cos from uh looking at it from my experience of being a patient, if you′re given a leaflet of a certain condition with a grey haired lady on the front, you don′t relate to them very well. See what I′m saying? So it's whether you′re buying into what you′ve been given. Even if you can, as you say, even if you can read it and understand the words. If you don′t think it's actually applicable because you decide that it's, you know?*

Moderator: *Can you see how someone could not know they′re got a heart problem but they are on a heart drug?*

Dr Jones: *Yes yes!*

Dr Gregory: *Very common.*

Dr Gregory: *‘Well I′ve had a heart attack and I′ve got better hence I haven′t got heart disease’.*

Nurse Rowan: *Or, ‘It was a bit irregular once but that's sorted out’.*

On the thorny issue of measuring HL and although only given brief attention by HP in these focus group discussions, there was a general (if not fully elaborated) dismissal of using an instruments to measure HL. In the context of the average ten minute appointment in UK primary care such measures were considered impractical due to lack of time and a perceived likely patient reluctance to reveal any literacy problems. Yet incomplete understanding or explanation of instructions was a common problem frustrating to patients and professionals. This is revealed in the following comment about lengthy appointment letters highlighting that communication can break down even before HP and patient meet leading to potential no-shows, underprepared and already confused patients:

*The appointment letter is two page and they never read the second page. They look at the second page when they get here when we point it out to say they should have bought a clean specimen of urine with them. And they say: ‘No, I never got asked to bring that’* (Mrs Robinson: FG6)

Patients, carers and professionals conceptualised and discussed health literacy at both the individual and system level. This key findings will be explored in further detail below. Analysis across the six focus groups found that participant understanding of health literacy could be fostered and facilitated as well as inhibited or challenged at both the system (or structural) and the individual (or agentic) level. The next section addresses participants' views on governance and responsibility for health literacy. Sub-themes are: interpersonal communication; intra-organisational communication and continuity of care; information management; and responsibility for health literacy.

### ii) Health literacy, governance and responsibility

#### Interpersonal communication

A problematic part of healthcare delivery and accessing information was poor inter-personal relationships. Patients identified the importance of communication, continuity and a sense of partnership or personal alliance that could be disrupted by both poor individual practice or by larger organisational obstacle. At the individual level approachable doctors who gave the appearance of having time and invited patients and carers to share in the consultation made a huge difference to how patients described that they felt about getting and comprehending the information they wanted. Something as simple as extra time for their routine appointments was very helpful:

*One doctor we have always used, when my husband went, always used to book a double appointment. And I invariably went in with him and he would look at me and say ‘Have you got any questions today’?* (Rosemary:FG2)

*Dr Chandler was fantastic. He made you feel you were the only patient in the world and discussed everything.* (Michael:FG2)

*There's many occasion [when] they′ve left the young doctors with us who has explained things and that's helped tremendously.* (Diane:FG3)

Continuity *within* a care sector facilitated good communication and as far as these patients were concerned, the key factor in good HL was effective communication and a relationship with their HP. In other words care could facilitate HL:

*I believe it starts with the GP. I′ve experienced both extremes and I had one GP who would say: ‘Oh yes, you′ve got this, take them’. [It] just was ‘get rid of you’. The GP I′ve got now is just the opposite: ‘Hello Christina, how are you, sit down, what's the problem’? And then he will tell me all about it and provide me literature on the problem. And you feel then that you want to know more. It's definitely beneficial having a good GP who explains things and makes you feel welcome.* (Christina:FG2)

*Well, if you have a good GP that is the answer. That can answer a whole lot of questions; you don′t need to go any further. [A good GP has] communication, interest, knowledge. Dr Sinclair and Dr Russell were both splendid … nothing was too much trouble for them. You know you felt they′d got all the time in world. You knew they hadn′t so you weren′t foolish enough to think you′d got them for half an hour, but they gave you that feeling that you were of great interest and they wanted to help you.* (Jennifer:FG1)

Stable organisational structures allowing patients to see familiar members of their health team facilitated these ‘caring’ relationships. However, what is also revealed is how evidently patient health literacy needs might vary, especially if explanations and information are poor or rushed. The issues of continuity and partnership in health, especially around comprehension and adherence to recommended advice could be both difficult to transmit or receive depending on a patient's emotional and physical state as these participants affirm:

*When you′re anxious you don′t remember things you may be told very clearly* (Jean:FG1)

*They′re also at the point of least being able to understand the information that they′re presented with because they′re most poorly* (Dr Gregory:FG5)

*People are so shocked it's like a mini bereavement when you′re diagnosed with rheumatoid arthritis* (Rosemary:FG2)

#### Intra-organisational communication and continuity of care

Continuity *between* care sectors was also regarded as essential to the promotion of health literacy and the experience of lack of continuity and fragmentation exemplified in both community and hospital settings was seen as deleterious to patient understanding, feeling cared for and ultimately use of health services. This concept emerged in the HP discussion too, whether this was because patients never saw the same doctor twice or (as a result of their complex and multi-faceted condition) on referral they saw several consultants from different specialities who did not seem to communicate with each other, or due to terse hospital letters. Lack of a holistic approach and continuity often led to negative clinical outcomes and patient dissatisfaction, typically arising from poor interpersonal and intra-organisational communication. Furthermore, both HP and patients had stories of professionals making erroneous assumptions about what information had previously been given to a patient particularly when these encounters has occurred in another care setting. Patients strongly believed that HP often could not appreciate properly the full range of challenges patients faced because of the wide-ranging impacts of their chronic conditions, or the long term impacts on them as individuals:

*I think it would help more if we saw the same person every time, if possible. Because you go in there and you think, ‘Well, do they know all about me?’ They haven’t had time to read all the information, and I think it would be good if people could see the same person each time.* (Michael:FG2)

This experience of fragmentation both between and within services and concomitant burden placed on individual HP, patients and carers was discussed in all focus groups. Everyone had experience or evidence of ‘departments not working together’ (Vivien:FG3). Primary care doctors were conscious of the communication void between the two sectors:

*[Patients] often come to ask us what happened and then we′ve got a [discharge summary] letter with three lines and we try to work out from that what actually happened in hospital* (Dr Gregory:FG5)

*For carers this could create a double burden leaving them feeling very isolated:*

*I just think everything has become so impersonal so far removed as to make you feel unimportant I suppose. Lost, that's probably another word you feel that you′re abandoned …you′re on your own that's how you feel you′re on your own.”* Diane FG4

However, an example of good practice in secondary care was mentioned in three different groups. It highlights how the practice of patient focused care could enhance patient understanding and sense of wellbeing. Here it is discussed in FG3:

*Vivien: Dr Evans dictates the letter that's going [to the GP] because if you hear anything you say, ‘Oh excuse me a moment’ and he stops dictating, doesn′t he? And he explains and then he sends you a copy of the letter that he's dictated which is fantastic. You′ve heard it once but, um, the words they use are not always lay words that we would use. He is brilliant like that*

*Flo: That must be very handy I′ve never had that happen*

*Keith: There's not many people do that, he does it to me too*

#### Information management

Most patients expected to supplement consultations with health professionals with their own endeavours such as searching out written material, media reports, anecdotal research and Internet searches. As the following extracts highlight, they recognised that the deluge of available information often is not helpful or relevant, they wanted to truly understand their condition not read unreliable opinions about it.

*[There are] some very good leaflets … they′re nice and simple and then you′ve got the internet which is the other extreme where you′ve got reams and reams and reams of it so somewhere in-between, you know. First of all you know you start off with the general knowledge and you move onto something a bit more (in-depth). You want to be able to find out how it actually affects you personally because we′re all different.* (Richard:FG1)

*I also see the other side, of the drivel printed every week [in the tabloid newspaper] which for most parts is very unhelpful at times shall we say.* (Keith, retired HP:FG3)

Similarly, GPs believed that it was their job to act as a conduit or filter to the ‘reams of information’ available to patients:

*We here get quite a lot of people who actually come with their reams [of information]. I think there are problems with information filtering as well as problems of informing people.* (Dr Shelley:FG5)

However, impersonal information sources alone were not adequate and patients were adamant that the relationship with HP was fundamental to understanding, specifically the caring relationship. Much effort in the HL field has to date gone into simplifying information and instructions. However, our data suggests patients and carers perceived the need for ‘layering’ of types information but with the key being not the information *per see* but it's delivery mode and context. David in FG1 sums it up as follows:

*Personally I think [a leaflet] should add to what you′ve been told it shouldn′t be instead of; it should be in addition to. So that if something is talked about in the consultation you can take away something and look at it which would then give you more information rather than expecting that information to take the place of the human interaction cos the piece of paper can′t look at you and see whether you′re stressed, whether it's worrying you.*

These (well engaged) patients tried hard to get expert advice and perspectives, and were mostly willing to accept the limitations of imperfect medical opinion as long as it did not become a barrier. The following exchange in FG2 exemplifies this and highlights a structural determinant governing access to healthcare in the UK where primary care doctors tend to be generalists, while specialists reside in the secondary care hospital setting and are accessed only after referral from primary care:

Rosemary: *So something you said that when you were talking to your GP and talking about your joints. And he basically told you to take pain killers. The GP might not know the difference between rheumatoid or osteo or whatever kind of arthritis*

Barbara: *Which I don′t know*

Rosemary: *He's not paid to know. He's paid to see there's a problem and refer you to someone who does know. I think sometimes the GP can be a brick wall, as well*

#### Shifting responsibilities in health literacy

This final sub-theme illustrates a significant debate that permeated these focus group discussions about whose responsibility it was to ensure patients understand and use healthcare services and treatment appropriately. Participants deliberated how roles and responsibilities had shifting from ‘doctor knows best’ to a situation today where patients were encouraged to be more autonomous. Despite the current political rhetoric in the UK to encourage responsibility and empowerment, older people in particular and for deeply ingrained cultural reasons were deemed to be far less likely to question the doctor or contribute to discussion about their health. Here are just two examples of this common debate:

*I think a lot of old people, especially in their 80's, those sort of age groups, the doctor was the saint and you don′t question him.* (Joan:FG2)

*They′re afraid you know afraid to ask the doctor because he's more important than they are, more educated than they are. Some don′t want to know any further than what's wrong and what tablets they′ve got give them, but I suppose I′m a bit inquisitive I like to know what things are all about.* (Deborah:FG1)

In terms of health literacy specifically, HP appeared to see the responsibility as theirs and spoke of patients at all levels of HL and their engagement (or lack thereof) with their own health management. The following extract between HP illustrates the complexity of the perceived struggle for patients (and professionals) with too much information, and a resistance to the expectation that they become medical experts:

Dr Shelley: *Maybe you′ve got to think about, when you take your car into the garage and they start gabbling on, you think: ‘I don′t care - just sort it’. Well, you′re not stupid, but you′ve got no idea what theymyre talking about.*

Nurse Rowe: *I′ve had quite a few people who [come back from hospital saying]: ‘They said I was to decide’. So then I tend to say to them, ‘Well, if they genuinely believed this then that is the emphasis they put and if it was genuinely a choice then that's why they′re saying it’. But people are feeling that healthcare professionals are a bit passing the buck.*

Dr Hunter: *Yes, telling them, ‘You decide’, and then not empowering them or giving them enough information.*

Patients did not advocate a paternalistic style of healthcare delivery, and were critical of peers with little interest in managing their own health. Nor did they advocate a delivery model which gave too much apparent choice or empowerment. Patients perceived that HL was just as much the responsibility of the patient as the professional but the HP had a responsibility to answer questions and impart information in such a way that enhanced health literacy:

*I had one doctor say ‘I wonder whether dispensing it prophylactically would help’ and I said ‘just remind me what prophylactically means’? And she said ‘oh I′m worrying you about something that might never happen’. And I said ‘never worry about telling patients what you′re thinking and what the options are’. We want to know, that helps us to work with you to get the best possible outcome. So I think we′ve got to play our part.* (Rosemary:FG2)

Furthermore, many patients saw it as a privilege of their role as consumers to choose if or when to be noncompliant. Enhanced health literacy gave them greater moral legitimacy to disagree with diagnosis or disregard recommended treatment:

*How things have changed in the last 20 well even in 5 years. There is so much that we can understand about what we′ve got and because we understand what we′ve got to a certain extent we can take responsibility for our own problems. And, well, not decide what drugs we′ll take but bend the rules may I say it a little bit.* (Jennifer:FG1)

Our patients and carers saw themselves as their own advocates and described querying details as part of their role especially with medication, including spotting mistakes, but said that this was a challenge for a lot of older people due to persisting cultural perceptions about the doctor:

*[What] patients ought to do is to question whether they still need to take a particular medicine or if you are on 3 or 4 things.* (Richard:FG1)

*Because they believe the doctors are right they believe the doctors are god more or less they think the doctors know everything and so they abide by them but I′ve had two or three occasions where mistakes have been made on my part and I′ve lost the centre of my eye because of failure and also given the wrong antibiotics by a doctor.* (Florence:FG3)

However, there was a sense that by taking responsibility and getting involved in decision making patients could potentially threaten their relationship with HP. In contrast, primary care professionals spoke about a recent shift from ‘offering choices’ or ‘option sharing’ with patients, to engaging patients in the management of their own care and ‘sharing the management’. Even so, there was a concern in both primary and secondary care from HP not knowing how much information it was appropriate to give. Concerns included frightening and/or confusing patients. Primary care HP spoke of their biases or preferences inevitably ‘creeping in’. Both patients and professionals described inhibitions relating to asking about understanding: both parties said that older patients could be reticent to ask questions – this was described as a ‘generational thing’; HP were anxious about asking if a person understood or could read. Asking about ‘illiteracy’ and even about understanding was compared to being as challenging as asking about sex.

## Discussion

Initially patients and professionals tended to interpret health literacy fairly narrowly, as compliance to medical treatment or self-directed research. They quickly expanded the concept, linking it to notions of empowerment through knowledge of their own conditions. Our patients, carers and professionals mostly saw HL not as an individual attribute or skill but rather as a result of interaction between patients and the healthcare system. Discussants agreed there was much scope for improved communication between patients and professionals. Patients expressed frustration at the perceived certainty that professionals knew more than they were saying; professionals voiced frustration that patients did not seem to have absorbed what professionals believed had been said or written down. Although patients praised support groups and certain practices, good face-to-face consultation experiences were the most valuable means of improving and maintaining HL. Despite limitations to our findings, patient perspectives were in some ways revelatory. Rather than dwelling on individual assets and skills patients saw HL as a *whole-system outcome*, highly dependent upon good communication, particularly in one-to-one consultations.

Health literacy research has placed emphasis on the individualised assets model while tending to overlook how to improve delivery. However, the need for a switch in emphasis has been advocated by others [26]–[28], and fits well with recent research on HL and shared decision making [29], [30]. That healthcare systems can form barriers to adequate health literacy – particularly in communication, and that professionals need to provide high quality care regardless of low HL has been recognised in practical guidance [10], [31]. A drawback of the traditional risk model is to see HL as prone to inevitable decline over time, especially as increasingly complex conditions develop in the adult lifespan. An alternative within the risk model perspective that allows for a dynamic understanding of HL is to focus on mitigating risk by improving both patient and professional assets (e.g. [32]). We suggest at the simplest level a *needs-*based understanding of HL is required. Just as patients have medical needs, they have needs in terms of skills, self-efficacy and for information to best manage their health related behaviour. These needs vary depending on social, cultural, temporal and medical context. A focus on HL needs can provide the foundations of many other proposed HL models. Rather than describe HL as a set of fixed patient assets across settings, perhaps it is a mismatch between variable assets and fluctuating needs that contributes highly to poor outcomes. A needs approach allows for change over time and for variations depending on patient, context, professional, disease and types of treatment – aspects particularly pertinent to ageing. Diverse approaches to health literacy needs and understanding patient roles have been suggested previously [33]. In practice many HL initiatives are run with more of a need than an asset based understanding. The simplistic functional-interactive-critical divisions for understanding HL [4] are inadequate for capturing the emotional needs and quite variable negotiation and communication skills of patients and professionals, particularly in a shared decision making environment. For instance, Smith et al found that some patients with nominally tested low functional literacy skills exhibited quite well-developed critical literacy skills, whereas some patients with tested high functional HL skills displayed relatively poor interactive or critical HL skills [34]. Other research highlights that patients often fail to fully engage in medical encounters because of perceived power imbalance or expectations that arise from previous social and cultural experiences [35], [36].

The needs-understanding of HL also promotes the value of personal relationships in medical care to make assessments, exchange information and teach skills. This might better be described as care rather than education and could fit better with the needs of an ageing population. These suggestions are sympathetic with Mol [37]. She argues that the apparent empowering model of *choice* is a poor focus in the delivery of healthcare and advice for individuals with chronic conditions. A *logic of choice* places an over-emphasis on empowerment of the patient, dismissing the emotional complexities of managing chronic illness, and the reality that even when individuals are highly informed and perfectly adherent, the nature and progress of their disease may still mean a poor outcome. Instead she advocates an emphasis on care rather than choice for patients with chronic conditions. Others have criticised excessive promotion of healthcare choices and the notion of personal independency or autonomy, arguing that what most patients would like and what would benefit them most, is not to decide for themselves the best treatment option, or to be compelled to go along with the choice determined by the health expert, but rather to know that the default and routine choice will mean excellent care without any need for them to personally apply critical analysis to various options [38], [39]. Although much data on health care quality and options are published and freely available in the UK (e.g., Care Quality Commission or National Institute for Clinical Evidence websites), there are large ethical questions about healthcare systems that expect so much of ill patients [20]. Significant inequalities may also arise because attributes such as knowledge are strongly linked to a patient's socio-economic status.

Nutbeam [4] suggests a deficiency in the HL risk model is its tendency to measure success mostly in terms of adherence. In reality, we argue that most health literacy interventions also consider improvements in awareness, skills and motivation. It should be possible to further widen ‘success’ in an HL intervention to include quality of communication, patient-led outcomes and quality of care. In applied research it is common to use adherence as a desirable outcome measure for an HL intervention. This is partly because adherence measures are assumed to be simple to consistently observe and describe, and because the relationship between health literacy and adherence is not well-understood, although it has been much discussed [16], [40]. Many health literacy interventions measure success very prominently in terms of adherence to recommended advice [e.g., [23], [41], [42]. As a result, the problems of non-adherence and low HL have become tangled and seemingly endemic despite widespread intervention efforts [43]. Instead, perhaps it should be seen as normal that for a single patient, both HL levels and adherence rates vary by condition and context (an idea also discussed in Nutbeam [44]). Thus it comes as no surprise that HL needs must be assessed continuously and can be expected to fluctuate in inconsistent ways, as it is the product of a joint experience between professionals and patients, each bringing individual and inconsistent skills and investment to the process.

### Limitations and lessons learned

Our participants had a specific interest in long term chronic (particularly musculoskeletal) conditions. Longer or repeat FGD might have elicited different perspectives on the value of HL in their own lives or work. Patients barely mentioned basic reading, numeracy or memory skills, although these deficits were a significant part of HP discussions. Patients could have been more explicitly invited to discuss any difficulties they or peers might have in very basic numeracy and communication skills. A different choice of video presentation might have led to different viewpoints, too.

The focus group patients and carers presented as a relatively knowledgeable group. We did not describe or formally administer an HL instrument, partly because it did not suit our aims of soliciting unguarded opinions. It might have been intimidating or insensitive. A reasonable alternative might have been to incentivise patients (with payment) to take a HL test, and later invite them to participate in FGD. Thus we might have had more success targeting patients whose functional HL levels were more clearly diverse. However, we cannot be sure that those who opted to participate were not still the most engaged and well-informed, or whose critical HL levels might have had little relationship with tested functional skills (following the experience of Smith et al [34]). Similarly, our health professionals were reluctant to ask patients about literacy or numeracy skills for fear of causing offence. HPs might have been less resistant if we had explained assessment methods that are not reading tests. We could have described ways to detect HL deficits using just a few qualitative and non-pejorative questions (eg [45]). However, we suspect that our HPs would still have worried about drawing conclusions from subjective answers as well as insufficient time available within routine appointments (typically around ten minutes with GPs [46]). Time is pressured for those providing secondary health care, too. The British National Health Service (NHS) is publicly funded, providing care for free at the time of use (no co-payments). The NHS is over-stretched in many areas and is widely considered to be under-resourced [47]. There are nonetheless strict targets that hospitals must meet regarding waiting times for patient appointments with many suggested negative consequences for other aspects of care [48]. Hence rapid delivery of service is foremost in the mind of health professionals. Support to assess health literacy is not a target or priority in the current system.

#### Recruitment Problems

We perceive that patients and carers were very engaged with healthcare management and were relatively health literate. As with all research recruitment, we are aware that our sample may not include hard to-reach individuals, including those who do not want to think about their health more than necessary. We suspect that some people are more attracted to the focus group format, while others find it intimidating, inconvenient or uninteresting. Given that engagement is a key ingredient to good HL, we should not be surprised that our FGD patients appeared to have relatively good HL skills. The assertive and engaged atmosphere highlights an inherent shortcoming in using FGD to identify important barriers to improving HL among those most at risk. It is hard to recruit patients with minimal skills or motivation, and previous research has highlighted issues of ‘shame’ associated with low HL [49], [50]. Some self-selection was inevitable and our findings cannot be said to be transferrable to the views of people with the most limited health literacy.

Low recruitment of carers was disappointing. It meant reduced input about HL in the context of providing for the needs of patients with cognitive or physical decline. HL skills among carers should be a particular concern in the context of ageing population profiles.

## Conclusions

It should be possible to integrate elements of both the assets and risk models of HL to produce something that better meets needs. A shift in professional and patient perspectives may be required to reemphasise quality of care rather than informed diversity of choice. The real challenge for qualitative research into HL may be to help clarify the useful purpose of the HL concept, why is it of interest and to whom. As a clinical risk factor it helps to identify communication and support needs. Beyond a fairly basic level, however, the value of the HL concept becomes less clear. It implies but yet is not the same as self-efficacy and empowerment [51], and yet success in HL promotion efforts is often measured in terms of adherence and outcomes. Patients with chronic conditions are increasingly encouraged to develop what might be called interactive or even critical HL skills, hopefully to increase patient autonomy. However, those skills may not increase confidence in recommended treatment. Perhaps not surprisingly in Smith et al. [34], patients invited to be critical of cancer screening options became less likely to choose any form of screening.

All of our participants seemed to prefer shared decision making rather than expect a norm of patient empowerment. We note that however resourceful and autonomous highly health literate patients such as ours might become, clinicians will customarily have more knowledge power due to the science-based nature of medicine [52]. Patients desire predictable interactions and search for more than knowledge, empowerment and responsibility from their engagement with healthcare. As Lupton [53] put it, we seek ‘affirmation and re-enactment of cultural, psychodynamic and affective processes (to make) everyday life choices, decisions and actions’. This attendant emotional based trust and desire for, at the very least, reciprocity in healthcare, is not an idealised engagement of two independent rational ‘health literate’ actors but rather an intersubjective interaction influenced by a variety of human needs and qualities.
